# Enhanced Sensitivity of Surface Acoustic Wave-Based Rate Sensors Incorporating Metallic Dot Arrays

**DOI:** 10.3390/s140303908

**Published:** 2014-02-26

**Authors:** Wen Wang, Xiuting Shao, Xinlu Liu, Jiuling Liu, Shitang He

**Affiliations:** State Key Laboratory of Acoustics, Institute of Acoustics, Chinese Academy of Sciences, No.21, BeiSiHuan West Road, Beijing 100190, China; E-Mails: shaoxiuting10@mails.ucas.ac.cn (x.S.); liuxinlu1987@foxmail.com (x.L.); liujiuling@mail.ioa.ac.cn (J.L.); heshitang@mail.ioa.ac.cn (S.H.)

**Keywords:** Coriolis force, delay line, metallic dot array, oscillator, SAW rate sensor

## Abstract

A new surface acoustic wave (SAW)-based rate sensor pattern incorporating metallic dot arrays was developed in this paper. Two parallel SAW delay lines with a reverse direction and an operation frequency of 80 MHz on a same X-112°Y LiTaO_3_ wafer are fabricated as the feedback of two SAW oscillators, and mixed oscillation frequency was used to characterize the external rotation. To enhance the Coriolis force effect acting on the SAW propagation, a copper (Cu) dot array was deposited along the SAW propagation path of the SAW devices. The approach of partial-wave analysis in layered media was referred to analyze the response mechanisms of the SAW based rate sensor, resulting in determination of the optimal design parameters. To improve the frequency stability of the oscillator, the single phase unidirectional transducers (SPUDTs) and combed transducer were used to form the SAW device to minimize the insertion loss and accomplish the single mode selection, respectively. Excellent long-term (measured in hours) frequency stability of 0.1 ppm/h was obtained. Using the rate table with high precision, the performance of the developed SAW rate sensor was evaluated experimentally; satisfactory detection sensitivity (16.7 Hz·deg·s^−1^) and good linearity were observed.

## 1. Introduction

Recently, there has been great interest in surface acoustic wave (SAW) rate sensors (so called gyroscopes) because of their many unique properties such as superior inherent shock robustness, a wide dynamic range, low cost, small size, and long working life compared to other current gyroscope types [1]. The Rayleigh wave can be generated at the surface of piezoelectric material by applying a voltage to interdigital transducers (IDTs) patterned on the substrate [2]. When the Coriolis force from the external rotation acts on the vibrating particles along the SAW propagation path, a pseudo running wave shifted by a quarter of a wavelength will be induced, that couples with the initial SAW generated by the IDTs, resulting in the change of trajectory of the wave particles, and hence, the acoustic wave displacement was deviated, leading to the acoustic wave velocity shift, the so-called SAW gyroscopic effect [3]. Consequently, a frequency variation proportional to the input rotation according to the relationship among the frequency was expected. Referring to the oscillator structure, the mixed oscillation frequency was used to evaluate the external rotation. Utilizing the so-called SAW gyroscopic effect, Lee *et al.* first realized a prototype of a micro rate sensor on ST quartz using the differential dual-delay-line oscillator configuration [4,5], temperature compensation was also conducted satisfactorily. Recently, some other meaningful research works concerning SAW gyroscopes were also reported [6,7]. In our previous work, a SAW gyroscope with similar structure based on a Y112°X LiTaO_3_ substrate was presented. It had a sensitivity of 1.332 Hz·deg^−1^·s over a wide dynamic range (0∼1,000 deg·s^−1^) and good linearity are obtained [8]. Obviously, the measured sensitivity is still far away from being useful in real applications.

**Figure 1.**
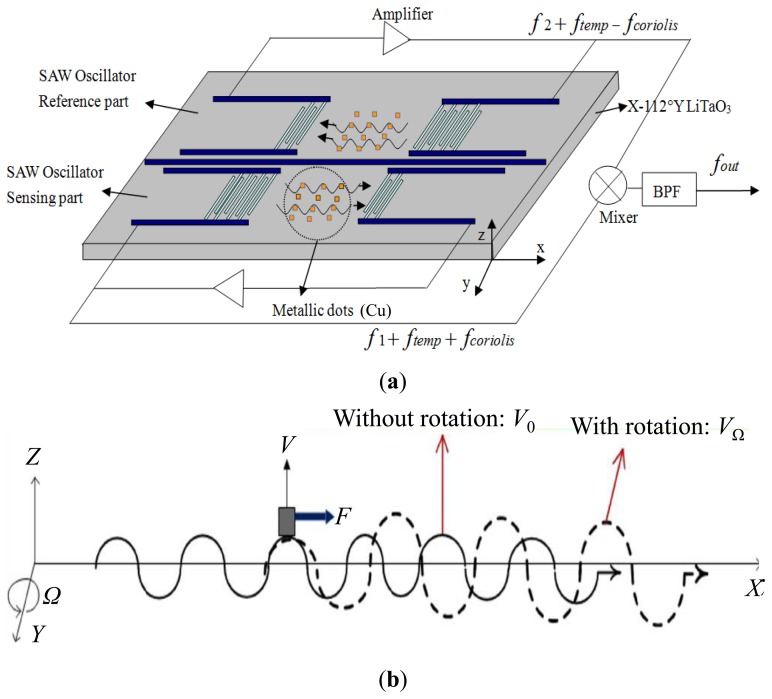
The schematic (**a**) and working principle (**b**) of the SAW based rate sensor with metallic dot array.

To improve the detection sensitivity, a creative idea was proposed herein whereby a metallic dot array was deposited strategically on the SAW propagation path of the SAW devices to enhance the Coriolis force acting on the propagating SAW [9]. A schematic and the working principle of such a rate sensor pattern is shown in Figure 1. A progressive SAW is generated between the IDTs of the SAW delay lines. Because the particle displacement of the Rayleigh wave has an out-of-surface motion that traces an elliptical path, the particles at the top and the bottom of the SAW vibrate normal to the surface and in the tangential direction, respectively. At the top and bottom of the progressive wave, metallic dots vibrate in the normal direction (±z axis) as shown in Figure 1b. When the sensor is subjected to an angular rotation, the Coriolis force acts on the vibrating metallic dots because of the Coriolis effect (*F*_coriolis_ = 2*m*(*v* × Ω); *m*: mass of dot, *v*: velocity of the dot, Ω: rotation rate). Moreover, the direction of the Coriolis force is the same as the direction of wave propagation. Therefore, the amplitude and velocity of the wave are changed, and this change induces a shift in the oscillation frequency of the oscillator. Additionally, to improve the detection sensitivity, a differential scheme was considered for the sensor configuration, that is, parallel and reverse settings are designed for two delay lines with metallic dot arrays, and the mixed oscillation frequency signal was used to characterize the applied rotation. Such differential scheme will double the sensitivity of the sensor and compensates the temperature effect [4], as mentioned in Figure 1. Despite the promising results, there still suffer from the lack of theoretical analysis on response mechanism and development of the prototypes of the sensor with metallic dot array.

Therefore, the first purpose of this paper is to establish the response mechanism of the SAW-based rate sensor incorporating a metallic dot array by using the approach of partial-wave analysis in layered media. Optimal design parametera such as the best metallic dot thickness was determined. Then, two SAW delay lines with SPUDT and combed transducer structures were designed and fabricated on a similar X-112°Y LiTaO_3_ wafer, acting as the feedback element of the oscillator for rate sensing, which is the second aim of this work. To improve the Coriolis force, a thick copper metallic dot array was deposited to the SAW propagation path of the SAW devices. Additionally, the developed two delay lines were set to in parallel and opposite direction. The X-112°Y LiTaO_3_ piezoelectric material was used as substrate because it is a good candidate for rate sensors due to its larger gyroscopic effect [2,10]. Then, using the frequency signal acquisition module, the mixed frequency shift from the oscillators was used to characterize the external rotation. And the performance of the developed SAW based rate sensor was evaluated experimentally by using the precision rate table.

## 2. Theoretical Model

**Figure 2.**
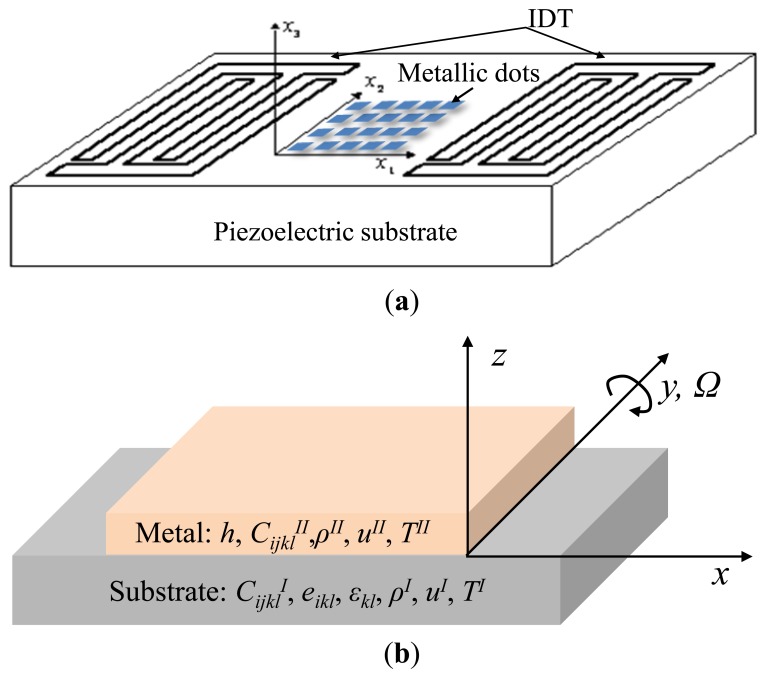
SAW device with metallic dot array used for gyroscope (**a**), and the coordinate system used in this study (**b**).

In this section, the pre-rotated SAW propagation considering the mass loading effect from the metallic dots along the SAW propagation path was analyzed theoretically by using the partial-wave analysis method [8,10]. A structure composed of a semi-infinite piezoelectric substrate (X-112°Y LiTaO_3_) and a metal layer over the substrate with thickness of *h* is constructed as shown in Figure 2.

### 2.1. Theoretical Model

As shown in Figure 2a, the SAW propagates along the *x*(*x*_1_) axis on the *x*-*y*(*x*_2_)-plane at *z*(*x*_3_) = 0. For the following analysis all material parameters of the medium are transformed into the coordinate system as shown in Figure 2b, like stiffness coefficients *C_ijkl_^II^*, density ρ^II^ for the metal layer; stiffness coefficients *C_ijkl_^I^*, the piezoelectric modules *e_ikl_*, the components of permittivity *ε_ik_*, and density *ρ*^II^ for the X-112°Y LiTaO_3_ substrate. First, the acoustic wave equation considering the contribution of Coriolis force in the metal layers is:

(1) $${C_{\text{ijkl}}}^{II}u_{k,jl}=\rho^{II}[{{\ddot{u}}_{i}}^{II}+2{ɛ}_{ijk}\Omega_{j}{{\dot{u}}_{k}}^{II}-(\Omega_{j}^{2}{u_{i}}^{II}-\Omega_{i}\Omega_{j}{u_{j}}^{II})],i,j,k,l=1,2,3$$

where Einstein's summation rule is used, and the indices changed from 1 to 3, *ρ^II^* is the density of the metal layer, *u_i_*^II^ is the component of the acoustic wave displacement. *ε_ijk_* is the Levi-civita symbol. The substrate in this paper is a piezoelectric medium whose only particle motion in the *x*_1_ and *x*_3_-direction is coupled with the electrical potential, so, only the components *u_1_* and *u_3_* was considered in the calculation. Additionally, in this paper, the SAW is assumed that propagates along the *x*_1_-direction, and attenuates in the *x*_3_-direction. Therefore, the solution of Equation (1) has the form as:

(2) $${u_{i}}^{II}={{\text{A}}_{i}}^{II}\exp[-\text{j}(\omega t-\beta x_{1}-\beta \eta x_{3}\left(\right]$$

where *A_i_* is normalized amplitudes, *η* is a decay constant, *ω* is the angular frequency. *β* = *ω*/*v* is the wave vector along the SAW propagation direction. Substituting the Equation (2) into Equation (1), the Christoffel equation in metal layer will be deduced as:

(3) $$\begin{array}{c} \Gamma \text{A}=0\\ \Gamma_{11}=c_{11}+2c_{15}\eta +c_{55}\eta^{2}-\rho_{1}v^{2}-\rho_{1}{\left(\Omega /\omega \right)}^{2}v^{2}\\ \Gamma_{13}=c_{15}+(c_{13}+c_{55})\eta +c_{35}\eta^{2}-j2\rho_{1}(\Omega /\omega )v^{2}\\ \Gamma_{31}=c_{15}+(c_{13}+c_{55})\eta +c_{35}\eta^{2}+j2\rho_{1}(\Omega /\omega )v^{2}\\ \Gamma_{33}=c_{55}+2c_{35}\eta +c_{33}\eta^{2}-\rho v^{2}-\rho_{1}{\left(\Omega /\omega \right)}^{2}v^{2}\\ \Gamma =\left(\begin{array}{cc} \Gamma_{11} & \Gamma_{13}\\ \Gamma_{31} & \Gamma_{33} \end{array}\right),A^{II}=\left(\frac{{\text{A}}_{1}}{{\text{A}}_{3}}\right) \end{array}$$

Only the determinant of coefficients was set to zero, so Equation (3) has a nontrivial solution, and gives an algebraic equation of the 4-th order in *η*. For a given value of SAW velocity *v* we obtain in general four eigenvectors *η_n_* (*n* = 1,2,3,4). Then, substituting the four eigenvectors into Equation (3), the corresponding normalized amplitude $A_{i}^{II(n)}$ can be determined. Full solution of the wave equation was the linear combination of four basic groups:

(4) $$\begin{array}{cc} u_{I}^{II}=\sum_{n=1}^{4}{c_{n}}^{II}{\text{A}}_{i}^{II(n)}\exp[-\text{j}(\omega t-\beta x_{1}-\beta \eta_{n}x_{3}\left(\right] & i=1,3 \end{array}$$

The coefficient *c_n_^II^* was determined by the boundary conditions. Next, we consider the SAW propagating in the piezoelectric substrate. The wave equation considering the Coriolis force contribution in substrate was:

(5) $$\left\{ \begin{array}{l} {C_{\text{ijkl}}}^{I}u_{k,jl}+e_{kij}\varphi_{,jk}=\rho^{I}[{u_{i}}^{I}+2{ɛ}_{ijk}\Omega_{j}{u_{k}}^{I}-\left(\Omega_{2}^{j}{u_{i}}^{I}-\Omega_{i}\Omega_{j}{u_{j}}^{I}\right)]\\ e_{jkl}{u_{k,jl}}^{I}-{ɛ}_{jk}\varphi_{,jk}=0 \end{array}\right.$$

The solutions of Equation (5) are represented as:

(6) $$\left\{ \begin{array}{l} {u_{i}}^{I}={A_{i}}^{I}\exp[-\text{j}(\omega t-\beta x_{1}-\beta \alpha x_{3}\left(\right]\\ \varphi ={A_{4}}^{I}\exp[-\text{j}(\omega t-\beta x_{1}-\beta \alpha x_{3}\left(\right] \end{array}\right.$$

Substituting Equation (6) into Equation (5), the Christoffel equation in the piezoelectric substrate can be obtained as:

(7) $$\begin{array}{c} \Gamma A^{I}=0\\ \Gamma =\left(\begin{array}{ccc} \Gamma_{11} & \Gamma_{13} & \Gamma_{14}\\ \Gamma_{31} & \Gamma_{33} & \Gamma_{34}\\ \Gamma_{41} & \Gamma_{43} & \Gamma_{44} \end{array}\right)A^{I}=\left(\begin{array}{c} A_{1}\\ A_{3}\\ A_{4} \end{array}\right)\\ \Gamma_{11}=c_{11}+2c_{15}\alpha +c_{55}\alpha^{2}-\rho^{I}v^{2}-\rho^{I}{\left(\Omega /\omega \right)}^{2}v^{2}\\ \Gamma_{13}=c_{15}+(c_{13}+c_{55})\alpha +c_{35}\alpha^{2}-j2\rho^{I}(\Omega /\omega )v^{2}\\ \Gamma_{14}=e_{11}+(e_{15}+e_{31})\alpha +e_{35}\alpha^{2}\\ \Gamma_{31}=c_{15}+(c_{13}+c_{55})\alpha +c_{35}\alpha^{2}+j2\rho^{I}(\Omega /\omega )v^{2}\\ \Gamma_{33}=c_{55}+2c_{35}\alpha +c_{33}\alpha^{2}-\rho^{I}v^{2}-\rho^{I}{\left(\Omega /\omega \right)}^{2}v^{2}\\ \Gamma_{34}=e_{15}+(e_{13}+e_{35})\alpha +e_{33}\alpha^{2}\\ \Gamma_{41}=\Gamma_{14},\Gamma_{43}=\Gamma_{34}\\ \Gamma_{44}=-{ɛ}_{11}-2{ɛ}_{13}-{ɛ}_{33}\alpha^{2} \end{array}$$

Similar to the analysis in the metal layer, the determinant of the coefficient is set to zero, and the six roots of the decay constant *α* can be obtained. We are only interested in the three eigenvectors with a negative imaginary part considering that the displacement and potential of the SAW must decrease with increasing depth into the substrate and vanish at infinity. Then, these roots are substituted into Equation (7), the corresponding three normalized amplitude $A_{i}^{I(n)}$ can be obtained, and the solution for the wave equation was:

(8) $$\left\{ \begin{array}{l} {u_{i}}^{I}=\sum_{n=1}^{3}{c_{n}}^{I}A_{i}^{I(n)}\exp[-\text{j}(\omega t-\beta x_{1}-\beta \alpha_{n}x_{3}\left(\right]\\ \varphi^{\Pi }=\sum_{n=1}^{3}{c_{n}}^{I}A_{4}^{I(n)}\exp[-\text{j}(\omega t--\beta x_{1}-\beta \alpha_{n}x_{3}\left(\right] \end{array}\right.$$

and the coefficient *c_n_^I^* was also determined by the boundary conditions.

To solve the above equations, we have to take in account the mechanical and electrical boundary conditions:

1. At the interfaces as substrate/metal layer), there should be continuity of stress and continuity of mechanical displacement:
  (9) $$\begin{array}{cc} u_{i}^{\text{I}}{|}_{x_{3}=0}=u_{i}^{\text{II}}{|}_{x_{3}=0},T_{i3}^{I}{|}_{x_{3}=0}=T_{i3}^{II}{|}_{x_{3}=0} & i=1,3 \end{array}$$
  where $\begin{array}{c} T_{i3}^{II}=c_{i3kl}^{II}u_{k,l}^{II} \end{array}$, $\begin{array}{c} T_{i3}^{I} \end{array}=c_{i3kl}^{I}u_{k,l}^{I}+e_{ki3}\varphi_{k}$.
2. At the top of the structure (free surface of the metal layer), there should be zero stress:
  (10) $$\begin{array}{cc} T_{i3}^{II}{|}_{x_{3}=h}=0 & i=1,3 \end{array}$$
3. Shorted circuit effect in the interfaces between the substrate and metal layer as:
  (11) $$\varphi {|}_{x_{3}=0}=0$$

Then, substituting the solutions of the wave Equations (4) and (8) into the boundary conditions Equations (9)–(11), the following equation can be obtained:

(12) $${\text{H}}_{m}{\text{C}}_{m}=0$$

The condition of nontrivial solution in Equation (12) was the determinant coefficient should be zero, that is:

(13) $$\left|{\text{H}}_{m}\right|=0$$

To simplify the theoretical calculation, the iteration method was used referring to the Matlab software. Based on the deduced formulas, the SAW velocity shift depending on the normalized rotation can be calculated.

### 2.2. Numerical Results and Discussion

**Figure 3.**
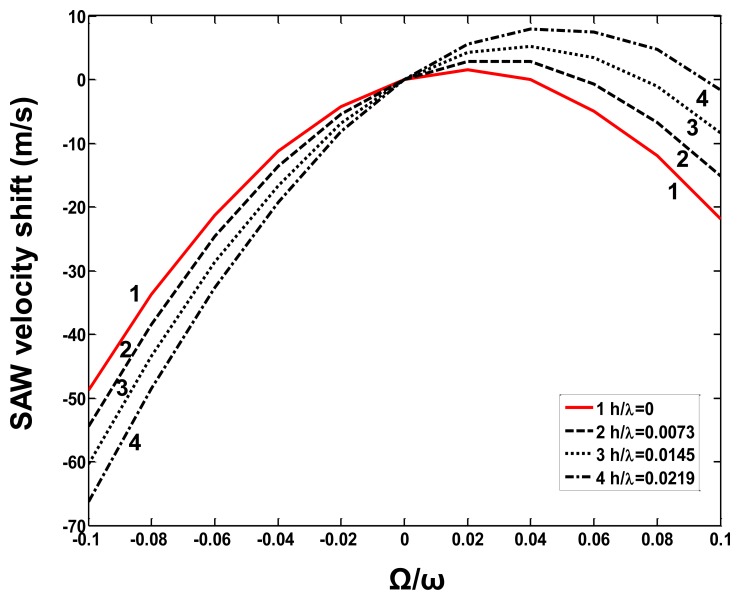
The SAW velocity shift depending on the normalized rotation in case Cu dots with various normalized thickness (h/λ).

**Figure 4.**
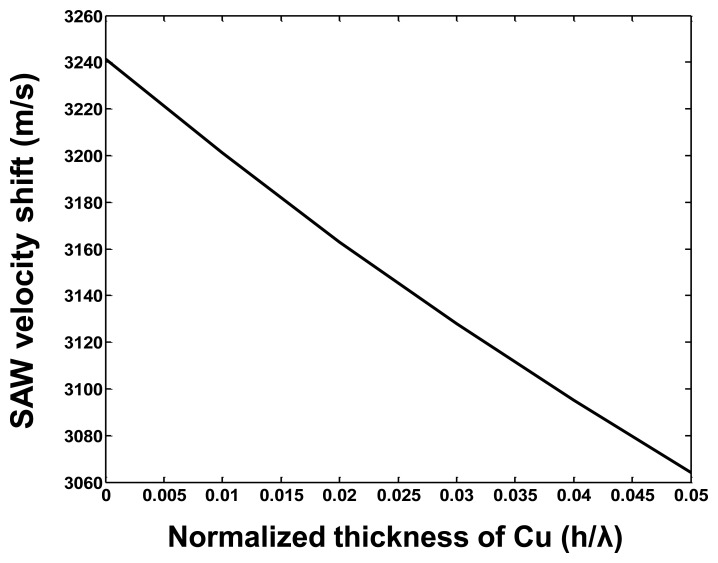
The SAW velocity shift depending on the normalized Cu thickness under given normalized rotation of 0.1.

In this paper, the pre-rotated SAW propagation on X-112°Y LiTaO_3_ substrate considering the mass loading contribution from the copper (Cu) dots distributed in the SAW propagation path is analyzed. The stiffness constants for Cu and X-112°Y LiTaO_3_ substrate, piezoelectric modules and permittivity constants of X-112°Y LiTaO_3_ substrate are listed in Table 1. The SAW velocity shift (*V_Ω_*–*V_0_*: *V_Ω_* is the acoustic velocity in pre-rotated status, and *V_0_* is the SAW velocity on the free surface) depending on the normalized rotation Ω/ω under a given Cu dot thickness was calculated as shown in Figure 3. The calculated results indicate that heavy metal materials like Cu lead to larger velocity shifts, that is, a larger sensor response will be obtained by using heavy meal dots, which is consistent with the experimental data, mentioned in [9]. Additionally, the effect of thickness of the metal dots on sensor response was also described, as shown in Figure 4. With the increase of the Cu thickness (normalized thickness from 0∼0.1), the velocity shift also decreases under a given normalized rotation of 0.1. However, a very thick Cu layer will result in a larger frequency deviation and higher attenuation, and also increased fabrication difficulty, so a balanced consideration should be done on the choice of Cu thickness. In our study, normalized thickness of ∼0.025 is advised, and as a result a satisfactory sensor response and lower wave attenuation are obtained.

## 3. Technique Realization

### 3.1. SAW Delay Line

**Figure 5.**
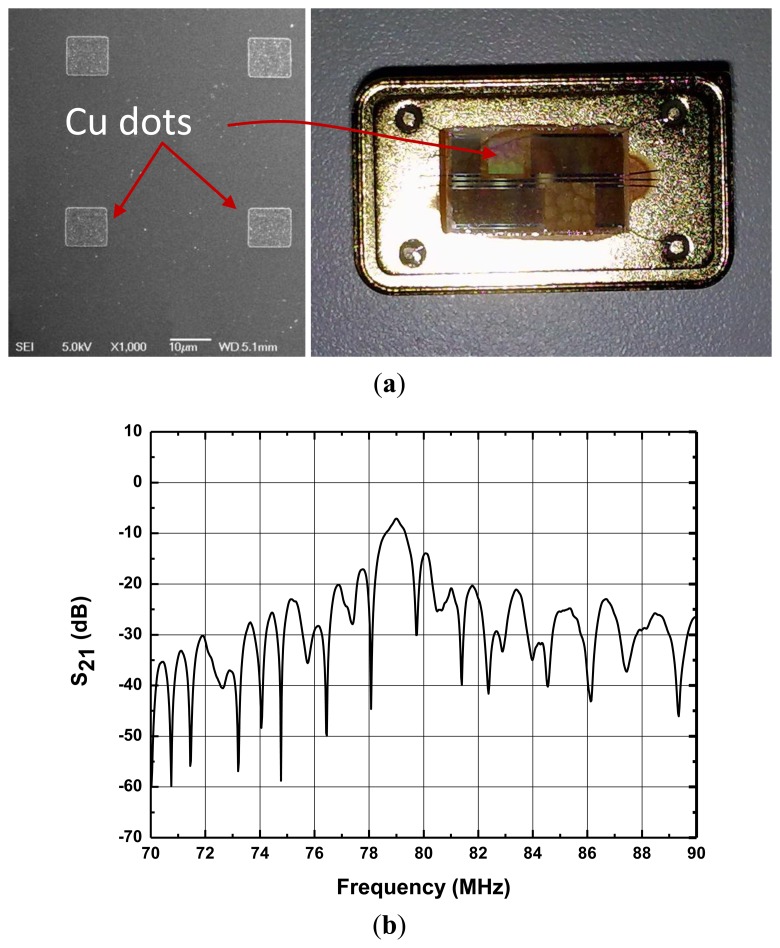
The fabricated SAW delay lines (**a**) and measured frequency response of the fabricated devices (**b**).

Two parallel SAW delay lines with opposite directions were fabricated by a photolithography technique on a same X-112°Y LiTaO_3_ substrate with Al metallization. The SPUDT and comb structure will improve the frequency stability of the oscillator effectively [13]. The SAW velocity on the X-112°Y LiTaO_3_ substrate with 110 nm Al metallization (*v_x_*) was 3,295 m/s. The operation frequency of the SAW delay line is specifically set to 80 MHz, thus, the wavelength λ of the SAW is 41.2 μm. Each delay line consists of a launching transducer and readout transducers, and the distance between the transducers was 68 λ. The length of launching transducer was set to 125 λ with four groups, which was about 80% of the center-to-center distance between the launching and readout transducers. In order to limit the total number of Al finger pairs in each transducer to about 20, the launching transducer was thinned into a comb structure. A large aperture of ∼1 mm (50 λ) was used. Copper metallic dot arrays of various thicknesses (100∼1,000 nm) were deposited onto the SAW propagation path of the SAW delay lines by a lift-off technique. The metallic dots are placed at the anti-node of the SAW. The size of each dot was designed to be λ_x_/4 and λ_y_/4 to reduce the effect of the metallic dot array on the SAW resonator, where λ_x_ and λ_y_ are the wavelengths along the x- and y-direction, respectively. The λ_x_ and λ_y_ are 41.2 μm and 39.3 μm, respectively, so the size of the Cu dots is 10.3 μm × 9.8 μm. The design of dot array was based on dot “unit cells”, each containing two dots. The spacing (center to center) of the basic unit cells is λ_x_ in x-axis and λ_y_ in y-axis [3]. Prior to the Cu deposition, a very thin Cr film was deposited for adhesion improvement. The fabricated dual delay line and SEM picture of the Cu dots is shown in Figure 5a.

Using a HP 8753D network analyzer, the amplitude and phase response of the SAW delay lines were measured under matched conditions, as shown in Figure 5b. A low insertion loss of 6.7 dB was observed from the developed SAW device with 900 nm Cu dots distribution.

### 3.2. SAW Oscillator

**Figure 6.**
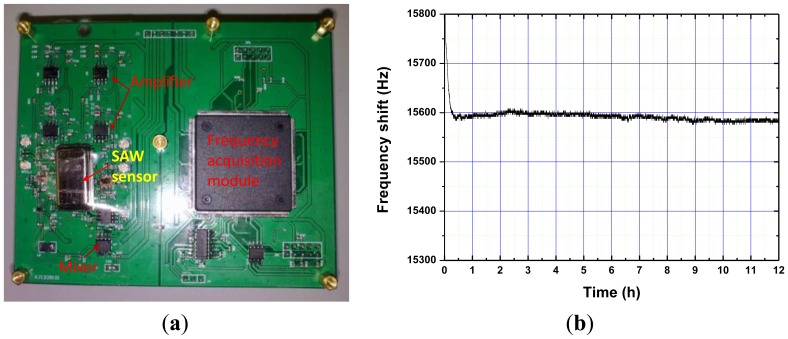
The fabricated SAW oscillator for rate sensor (**a**) and frequency stability measurement of the oscillator (**b**).

Next, the fabricated SAW device chip was loaded onto a standard metal base (Figure 5a), and acted as the oscillator feedbackf. The launching and readout transducers of the fabricated SAW delay lines were connected by an oscillator circuit which was made up of discrete elements (amplifier with a gain of 25 dB, phase shifter, mixer and LPF and so on) on a printed circuit board (PCB) as shown in Figure 6a. The output of the amplifiers were mixed in order to obtain a difference frequency in the kHz range. This technique allows doubling of the detection sensitivity and reduction of the influence of the thermal expansion of the substrate. The sensor response was picked up by the frequency acquisition module on the PCB and output to a PC. It is well-known that the frequency stability of the oscillator directly affects the threshold limit of detection and stability of the sensor. Thus, an experiment was performed to evaluate the frequency stability of the fabricated SAW oscillator at room temperature (20 °C). Also, the oscillation was modulated at the frequency point with lowest insertion loss by a strategically phase modulation [13]. The typical long-term stability (*h*) of the oscillator was measured as 0.1 ppm/h (Figure 6b).

## 4. Sensor Experiments and Discussions

**Figure 7.**
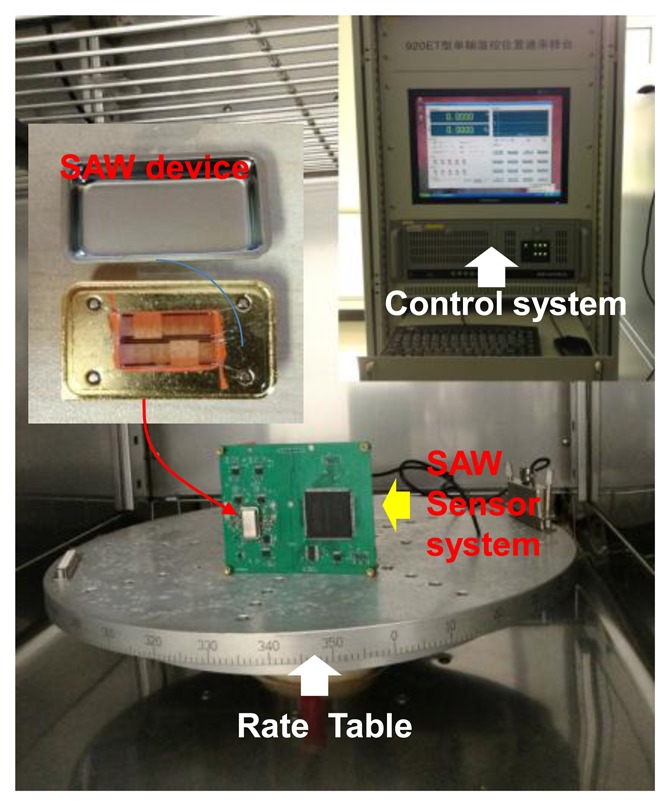
The measurement setup of the developed SAW rate sensor.

Then, the packaged SAW sensor was mounted onto the PCB board, and the performance was evaluated experimentally. The experimental apparatus setup for the performance evaluation was composed of a precision temperature-controlled rate table (Figure 7) and a PC for recording the sensor responses.

**Figure 8.**
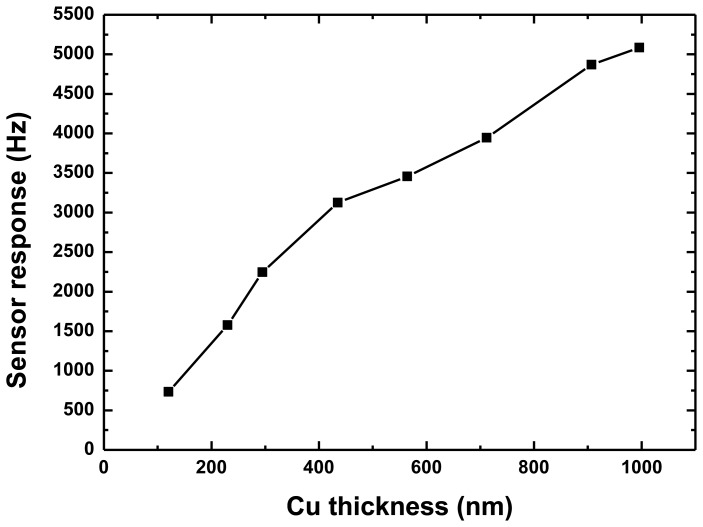
Measured sensor response depending on thickness of Cu dots in case rotation of 300 deg/s was applied.

The PCB board with packaged SAW sensor was placed on the rate table in a temperature-controlled chamber. An input voltage of +5 V was applied to the PCB. The frequency response of the developed sensor varies with the applied external rotation in a systematic fashion. First, an experiment was performed to validate the effect of the Cu dot thickness on the sensor response, as shown in Figure 8. With the increase of the thickness of the Cu dots, the frequency response towards a given rate of 300 deg/s increases along with it, which is consistent with the theoretical predictions mentioned in Figure 4. However, a very thick Cu dot will lead to increasing technical difficulty. Additionally, the increasing insertion loss of the SAW delay lines results in deterioration of the frequency stability of the oscillator, so the thickness of the Cu dots was considered as 900 nm (normalized thickness of ∼0.022) in our work.

**Figure 9.**
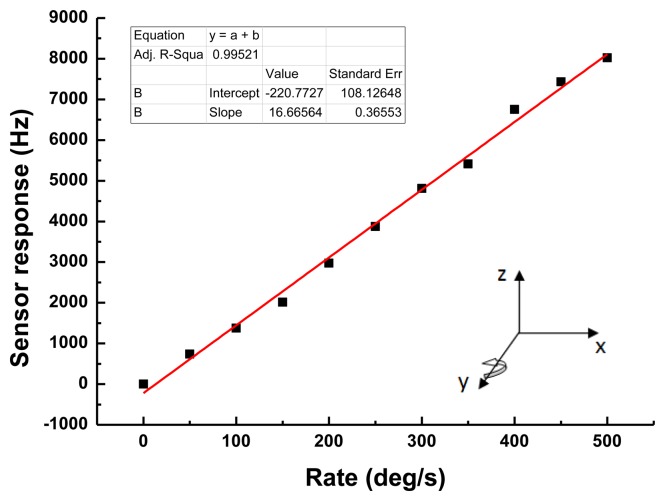
The sensitivity evaluation of the present SAW rate sensor.

Then, the detection sensitivity of the developed SAW based rate sensor with 900 nm Cu dots distribution was evaluated, as shown in Figure 9. A very clear frequency shift depending on the applied rotation was observed in the rate range of 0∼500 deg/s. The sensitivity and linearity of the sensor with rotation in the *y*-axis were evaluated as 16.7 Hz·deg^−1^·s and 0.99, respectively. The measured sensitivity is over 12 times larger than that of reported similar gyroscopes [8].

## 5. Conclusions

A new SAW based rate sensor on X-112°Y LiTaO_3_ with an operation frequency of 80 MHz was developed, and it was composed of two SAW delay line oscillators with opposite direction. To improve the Coriolis force, a copper metallic dot array was deposited onto the SAW propagation path of the SAW devices. The partial-wave analysis in layered media approach was used to analyze the response mechanism and determine the design parameters. SPUDT and combed transducers were used to structure the SAW delay line to minimize the insertion loss and improve the frequency stability of the oscillator. The developed SAW based rate sensor was evaluated using a precision rate table. Sensitivity of 16.7 Hz·deg^−1^·s in testing range of 0∼500·deg·s^−1^, and good linearity were obtained.

## Figures and Tables

**Table 1.** The mechanical parameters of the substrate and the metal layers [11,12].

**Materials**	**Stiffness Coefficients** **(10^10^ N/m^2^)**	**Piezoelectric Modules** **(C/m^2^)**	**Permittivity Constants** **(10^−12^ F/m)**	**Density** **(kg/m^3^)**
X-112°Y LiTaO_3_	C_11_: 23.3 C_33_: 27.5 C_44_: 9.4 C_12_: 4.7 C_13_: 8.0 C_14_: −1.1	e_15_: 2.58 e_22_: 1.59 e_31_: −0.24 e_33_: 1.44	ε11: 51 × ε_0_ ε33: 43 × ε_0_ ε_0_: 8.854	7,450
Copper	C_11_: 17.69 C_33_: 7.96			8,900
